# Cardiovascular adjustments during anticipated postural changes

**DOI:** 10.14814/phy2.13554

**Published:** 2018-01-15

**Authors:** Neesirg M. Patel, Ethan A.G. Baker, Samuel R. Wittman, Isaiah C. Engstrom, George H. Bourdages, Andrew A. McCall, Derek M. Miller, Bill. J. Yates

**Affiliations:** ^1^ Department of Otolaryngology University of Pittsburgh Pittsburgh Pennsylvania

**Keywords:** Anticipation, cerebral blood flow, conditioned cardiovascular response, orthostatic hypotension, sympathetic nervous system

## Abstract

It is well‐documented that feedforward cardiovascular responses occur at the onset of exercise, but it is unclear if such responses are associated with other types of movements. In this study, we tested the hypothesis that feedforward cardiovascular responses occur when a passive (imposed) 60° head‐up tilt is anticipated, such that changes in heart rate and carotid artery blood flow (CBF) commence prior to the onset of the rotation. A light cue preceded head‐up tilts by 10 sec, and heart rate and CBF were determined for 5‐sec time periods prior to and during tilts. Even after these stimuli were provided for thousands of trials spanning several months, no systematic changes in CBF and heart rate occurred prior to tilts, and variability in cardiovascular adjustments during tilt remained substantial over time. We also hypothesized that substitution of 20° for 60° tilts in a subset of trials would result in exaggerated cardiovascular responses (as animals expected 60° tilts), which were not observed. These data suggest that cardiovascular adjustments during passive changes in posture are mainly elicited by feedback mechanisms, and that anticipation of passive head‐up tilts does not diminish the likelihood that a decrease in carotid blood flow will occur during the movements.

## Introduction

The cardiovascular responses necessary to maintain homeostasis during postural alterations have been well‐described for both humans and animals (Blomqvist and Stone 1983; Yates et al. 2014; Ricci et al. 2015). A variety of sensory inputs, including those from baroreceptors and vestibular receptors, contribute to triggering alterations in sympathetic nervous system activity during changes in body position in space (Yates et al. 2014). Inadequacy of these homeostatic responses can result in orthostatic hypotension (Johnson 1976; Stewart 2012). Postural changes often occur as a component of voluntary movement, but can also be imposed, as during tilt table testing in the laboratory or falls and vehicular travel in the real world.

A variety of studies have shown that exercise‐related cardiovascular responses are initiated in a feedforward fashion, synchronous with the onset of physical activity (Waldrop et al. 1996; Fadel and Raven 2012; Fisher et al. 2015). Goodwin, McCloskey, and Mitchell coined the term “central command” to describe these feedforward cardiovascular responses during exercise (Goodwin et al. 1972). Gandevia et al. (1993) provided definitive evidence for the existence of central command by showing that paralyzed human subjects exhibited increases in heart rate and blood pressure when they imagined exercising. There is also evidence for central command in animals. Rats conditioned to run on a treadmill showed increases in heart rate, blood pressure, and muscle blood flow before exercise was initiated, just after they were placed on the treadmill (Armstrong et al. 1989). Other experiments demonstrated that stimulation of a variety of regions in the midbrain or diencephalon of anesthetized or decerebrate animals produced parallel cardiovascular and motor responses (Waldrop et al. 1996; Nakamoto et al. 2011; Matsukawa 2012).

However, it is less clear whether feedforward cardiovascular responses occur prior to and during movement paradigms other than exercise, such as head‐up postural changes that result in diminished venous return of blood to the heart. Smith et al. (2000) reported that baboons did not routinely exhibit changes in heart rate, blood pressure, or renal blood flow prior to standing, but the data in this study were limited and compromised by difficulties in synchronizing body position determined from videotape recordings with recordings of cardiovascular parameters obtained using telemetry. In contrast, studies in humans showed that illusory tilts could elicit changes in heart rate and blood pressure, raising the prospect that expectation of postural changes may elicit cardiovascular responses (Wood et al. 2000).

This study used a feline model to test the hypothesis that feedforward cardiovascular responses occur when a 60° head‐up tilt is anticipated, such that changes in heart rate and carotid artery blood flow (CBF) commence prior to the onset of the rotation. We additionally used a classical conditioning approach in which 20° tilts were substituted for a subset of 60° tilts. As animals expected a 60° tilt during these “false cued” 20° trials, we tested the hypothesis that the 20° tilts would evoke similar changes in heart rate as the anticipated 60° tilts, and that the cardiovascular responses elicited in the absence of the predicted orthostatic challenge would cause CBF to increase.

## Methods

All experimental procedures conformed to the *Guide for the Care and Use of Laboratory Animals* (National Research Council 2011), and were approved by the University of Pittsburgh's Institutional Animal Care and Use Committee. Data were collected from seven purpose‐bred adult cats of either sex, with characteristics indicated in Table 1. Animals were obtained from Liberty Research, Inc. (Waverly, NY). Animals received commercial cat chow and water ad libitum, and were housed under a 12‐h light/12‐h dark light cycle.

**Table 1:** Information about animals and parameters of the study

Animal	Gender	Weight at surgery (kg)	Weight at end of data collection (kg)	Total period of data collection (days)	Period of data collection before 20° tilts introduced (days)	Total number of 60° trials	Total number of 20° trials
1	Female	2.5	3.5	95	45	1033	88
2	Male	4	6	201	64	2529	71
3	Male	3.4	4.5	52	36	963	32
4	Female	3	5	172	46	2143	171
5	Female	5	6	128	63	2138	71
6	Male	3.1	3.4	15	a	262	a
7	Male	5	5.1	31	a	570	a

a False‐cued 20° tilts were not performed in this animal.

### Experimental overview

We made use of a feline model in these experiments, as cats have a long longitudinal axis and are subject to pronounced redistributions of blood volume during head‐up tilts (Yavorcik et al. 2009). In addition, cats can be acclimated for restraint on a tilt table during experimental sessions lasting over an hour (Jian et al. 1999; Cotter et al. 2001, 2004; Holmes et al. 2002; Mori et al. 2005; Wilson et al. 2006a,b; Arshian et al. 2017; Yavorcik et al. 2009). A bright light cue was presented to animals in a darkened room to signal that a tilt was imminent. Such light stimuli have often been used in conditioning experiments in animals (Schenk and Partridge 2001; Chess et al. 2005; Martin‐Garcia et al. 2009; Itzhak et al. 2010), as they are highly salient without producing startle.

**Figure 1:**
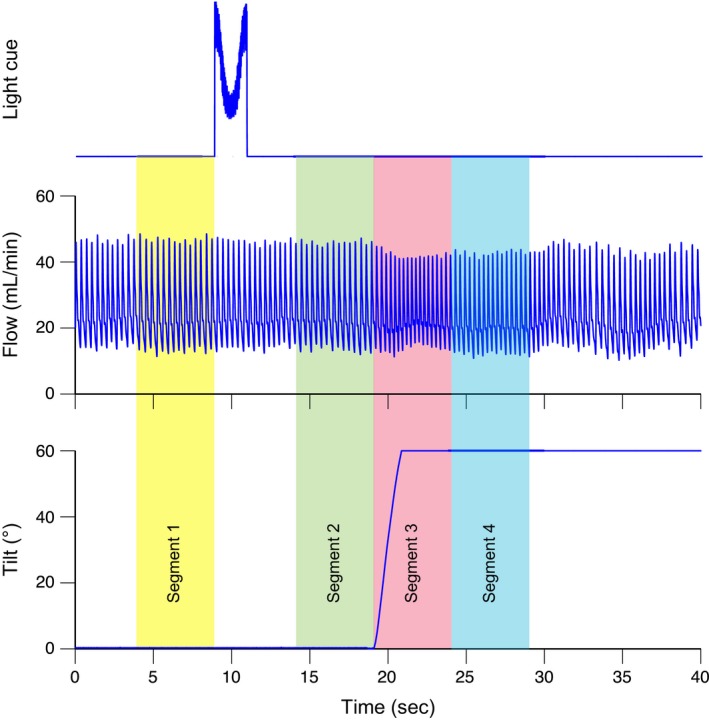
Example of data collected in experiments. Top trace: signal from photometer mounted on the tilt table, indicating the timing of the light cue; Middle trace: recording of pulsatile CBF; Bottom trace: signal from tilt table potentiometer, reflecting table position. Shading shows the four time periods considered for each tilt. Segment 1: 5‐sec period prior to the light cue. Segment 2: 5‐sec period prior to tilt onset (and following the light cue). Segment 3: 5‐sec period commencing at tilt onset. Segment 4: subsequent 5‐sec period when the animal was positioned head‐up.

Animals were slowly acclimated over 18–70 days for restraint in a cat restraint bag, which was attached using straps to a tilting device, as in previous studies (Jian et al. 1999; Cotter et al. 2001, 2004; Holmes et al. 2002; Mori et al. 2005; Wilson et al. 2006a,b; Arshian et al. 2017; Yavorcik et al. 2009). Subsequently, the surgical procedure described below was performed to place perivascular probes (PS series, Transonic Systems, Ithaca, NY) around the common carotid arteries and secure a bolt to the skull to permit head restraint. Another period of acclimation for body and head restraint (by placing a screw into a head‐mounted bolt) occurred over 27–104 days until animals remained sedentary without vocalization for a period of 1 h. Data recording then commenced, typically every weekday with the exception of holidays. The testing room was dimly illuminated throughout experimental sessions. During each recording session, ~30 head‐up tilts were delivered, each preceded by a bright (400 lumen) light cue of 2–sec duration starting at 10 sec before the onset of the tilt (see Fig. 1). This timing interval between the cue and stimulus was selected, as we previously found that reflex‐elicited cardiovascular adjustments occur at 5–10 sec after tilts (Jian et al. 1999; Holmes et al. 2002; Mori et al. 2005; Wilson et al. 2006a,b; Yavorcik et al. 2009). The light cue was provided by a remotely controlled grid of nine red and nine blue LEDs (Kick light, Rift labs, Boston, MA) placed ~2.5 m directly in front of the animal's face. During the cue, all red LEDs were illuminated while the blue LEDs flashed (250 msec on/250 msec off). Animals were maintained in the head‐up position for ~30‐sec, prior to being returned to the prone (baseline) position. Tilts were separated by ~60 sec. Carotid blood flow (CBF) was monitored during recording sessions; heart rate was determined from peaks in CBF. After 60° tilts had been delivered for 36–64 days, during ~10% of trials a 20° tilt was pseudorandomly substituted for the 60° tilt in five of the animals (see Table 1). The data collection period was 15–201 days, and was discontinued when pulsatile CBF disappeared, presumably as a consequence of the perivascular probe becoming detached from the carotid artery.

### Surgical procedures

An aseptic surgery was performed on each animal in a dedicated operating suite. Animals were initially anesthetized by an intramuscular injection of ketamine (20 mg/kg) and acepromazine (0.2 mg/kg). An endotracheal tube was inserted, and anesthesia was continued using 1–2% isoflurane vaporized in O_2_. Pulse oximetry was used to assure that heart rate and blood oxygenation remained stable during the procedure; vital signs (heart and respiration rates; blood oxygen saturation) were recorded in the animal's anesthesia record at least every 15 min. An intravenous catheter was also inserted, and ringer lactate solution was infused to replace fluid loss during the surgery. A heating pad and heat lamp were used to maintain core temperature near 38°C.

Longitudinal incisions were made on the ventral surface of the neck on each side of the trachea. Blunt dissection through the sternothyroid and sternohyoid muscles provided access to the common carotid artery on each side. Care was taken not to disturb surrounding tissues while a perivascular probe was placed around each carotid artery and secured in place with sutures. The cable from each probe was routed subcutaneously to the dorsal aspect of the skull, and the muscle and skin overlying the probes were closed using sutures. The dorsal skull was subsequently exposed, and a head fixation bolt and the probe connectors were secured to the skull using dental cement. Following surgeries, amoxicillin (50 mg oral) was administered twice daily for 10 days and transdermal fentanyl (25 g/h, Jannsen Pharmaceutical Products, Titusville, NJ) was provided for 3 days.

At the conclusion of data collection, animals were anesthetized using intramuscular ketamine (20 mg/kg) and acepromazine (0.2 mg/kg) followed by intraperitoneal pentobarbital sodium (40 mg/kg) and transcardially perfused with 10% formalin.

### Data collection procedures

After animals were acclimated for restraint on the tilt table, data collection was initiated. During recording sessions, a cable was used to connect each perivascular probe to a Transonic Systems TS420 perivascular flow module, which provided instantaneous volume flow measurements. A potentiometer and photodetector mounted on the tilt table were used to monitor table position and the light cue provided prior to tilts, respectively. Signals from the flow modules, potentiometer, and photodetector were recorded digitally at 100 Hz using a Cambridge Electronic Design (Cambridge, UK) 1401‐plus data collection system and Spike‐2 software running on a Dell computer.

The tilt table was rotated manually and secured in the tilted position using a locking device that permitted movement to one of the three predetermined tilt positions. The individual manipulating the tilt table was positioned behind the animal, and monitored the animal for movement and vocalization during the trials. A second individual was responsible for data collection and logging, as well as triggering the light cue. This second individual provided a verbal countdown to the person operating the tilt table, to assure that timing of the light cue and onset of the tilt were precise. The countdown could have served as an ancillary conditioned stimulus. After each trial, the presence of animal movement or vocalization were recorded in the laboratory record.

### Data analysis procedures

Spike‐2 data files were imported into Matlab (Mathworks, Natick, MA) for analysis. For each trial, the onset time of the light cue was determined, as were the time of the onset and peak of the tilt following the cue. From these determinations, four 5‐sec segments of each trial were designated for analysis, as indicated in Figure 1: the period prior to the light cue *(segment 1)*; the period immediately prior to tilt onset *(segment 2)*; the period following the onset of the tilt, which included the table movement *(segment 3)*; the subsequent period while the animal was in the head‐up position *(segment 4)*. For each of these periods, average CBF and the average latency between each peak in CBF were determined; the latter measure was used to determine heart rate in each segment. The time between the light cue and tilt onset were also established, as were the duration and velocity of the tilt.

Statistical analysis and plotting of experimental results were conducted using Prism 7 software (GraphPad Software, La Jolla, CA). Confidence intervals are indicated as mean ± SD. Trials in which the average tilt velocity varied from the overall mean (36°/sec) by two or more standard deviations (12°/sec) were excluded from data analysis, as were any trials in which the latency between onset of the light cue and tilt onset was outside of the acceptable range of 9–11 sec. The average period between the onset of the light cue and a 60° tilt was 10.2 ± 0.5 sec, and the average period between the onset of the light cue and a 20° tilt was 10.3 ± 0.6 sec. In addition, trials were excluded if any animal movement or vocalization occurred. Only 9.8% of trials were excluded for any of these reasons.

## Results

**Figure 2:**
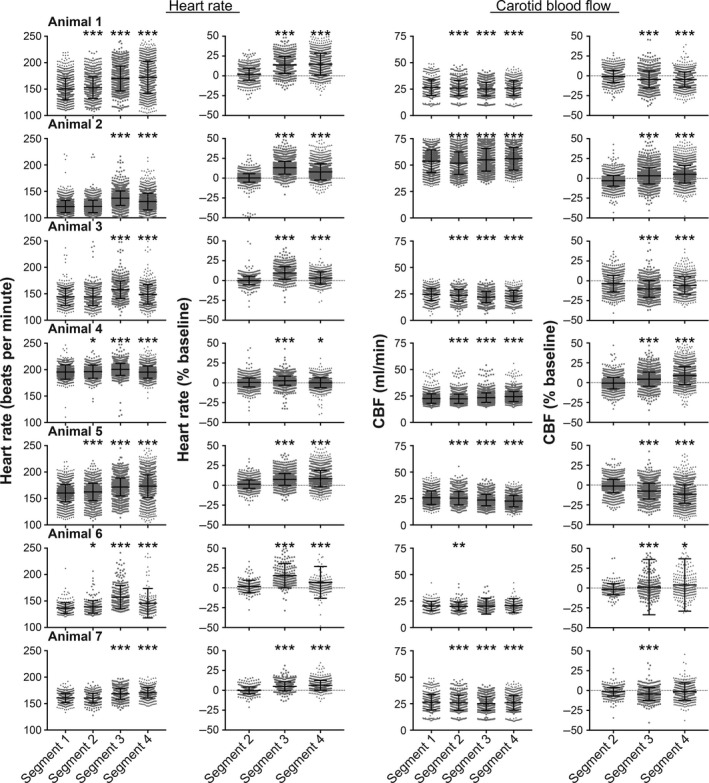
Changes in heart rate (left side) and CBF (right side) that occurred during every 60° tilt in each animal. Data for the four recording segments (see Fig. 1) are provided separately; the total number of 60° tilts performed for each animal is indicated in Table 1 (in the column “total number of 60° trials”). For designations of heart rate and CBF, the left column indicates raw values, whereas the right column provides the percent changes in values in segments 2, 3, and 4 from those in segment 1 (baseline). Symbols above each group of symbols designate whether the average value is significantly different (using a one‐way ANOVA analysis with Bonferroni correction) from that for the leftmost column of each panel (either baseline heart rate or CBF in segment 1 [left columns] or percent changes from baseline in segment 2 [right columns]). *, *P* < 0.05; **, *P* ≤ 0.001; ***P ≤ 0.0001. This figure shows that responses varied considerably from trial‐to‐trial, and were larger in segments 3 and 4 than in segment 2.

**Figure 3:**
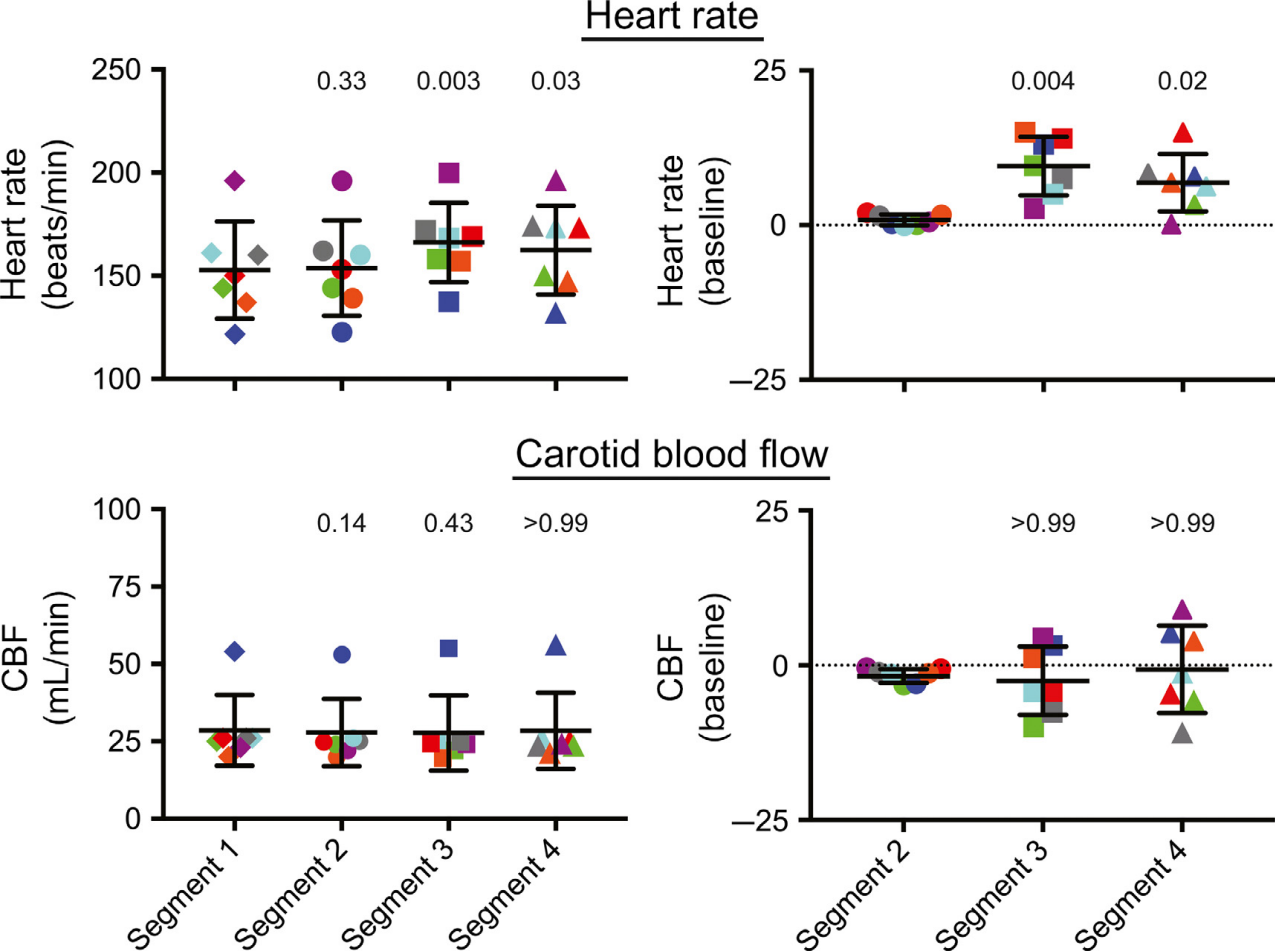
Average percent changes in heart rate and CBF during 60° head‐up tilts in the seven animals (the average value for a recording segment in each animal is designated as a single data point). Heart rate is shown in the top panels, while CBF is indicated in the bottom panels. The left panels indicate raw values, whereas the right panels provide the percent changes in values in segments 2, 3, and 4 from those in segment 1 (baseline). Numbers above each group of symbols are *P*‐values (from a one‐way ANOVA analysis with Bonferroni correction) designating whether the average value is significantly different from that for the leftmost column of each panel (either baseline heart rate or CBF in segment 1 [left panels] or percent changes from baseline in segment 2 [right panels]). Error bars designate one standard deviation. Data for each animal are designated by symbols of different colors: animal 1, red; animal 2, dark blue; animal 3, green; animal 4, purple; animal 5, gray; animal 6, orange; animal 7, light blue. These findings show that heart rate increased during 60° head‐up tilts, but that CBF could either increase or decrease during tilts.

Figure 2 shows the changes in heart rate and CBF that occurred during every 60° tilt in each animal. Figure 3 shows the pooled data from all animals (the average value for a recording segment in each animal is designated as a single data point). In every animal, heart rates were significantly higher (one‐way ANOVA analysis with Bonferroni correction) in segment 3 than in segment 1, and in all but one animal (animal 4) heart rates were significantly higher in segment 4 than in segment 1. Accordingly, an analysis of pooled data from all animals (Fig. 3) confirmed that heart rates were higher (*P* < 0.05) during segments 3 and 4 than during segment 1. In four of the seven animals (animals 1, 4, 5, and 6), mean heart rate was slightly elevated in segment 2 over that in segment 1. However, the analysis of pooled data from all animals (Fig. 3) failed to confirm that heart rate was systematically elevated in segment 2 over baseline values. In addition, when percent changes in heart rate from baseline were compared for segments 2, 3, and 4 (one‐way ANOVA analysis with Bonferroni correction), the increases in heart rate in segments 3 and 4 were shown to be larger than in segment 2 in every animal (Fig. 2), as well as the pooled analysis (Fig. 3).

Figure 2 also shows that CBF changed significantly during tilts in most of the animals (with the exception of animal 6). However, in some cases blood flow increased slightly (animals 2 and 4) while in others it decreased (animals 1, 3, 5, and 7). Consequently, when the average CBF data from all animals were considered (Fig. 3), no significant changes were noted. Some small variations in blood flow from baseline occurred in segment 2 (after the light cue but prior to tilts), but the average magnitude was small (1.8 ± 1.1%). Moreover, in every case but animal 6, the change in blood flow during segment 3 was significantly larger than in segment 2.

**Figure 4:**
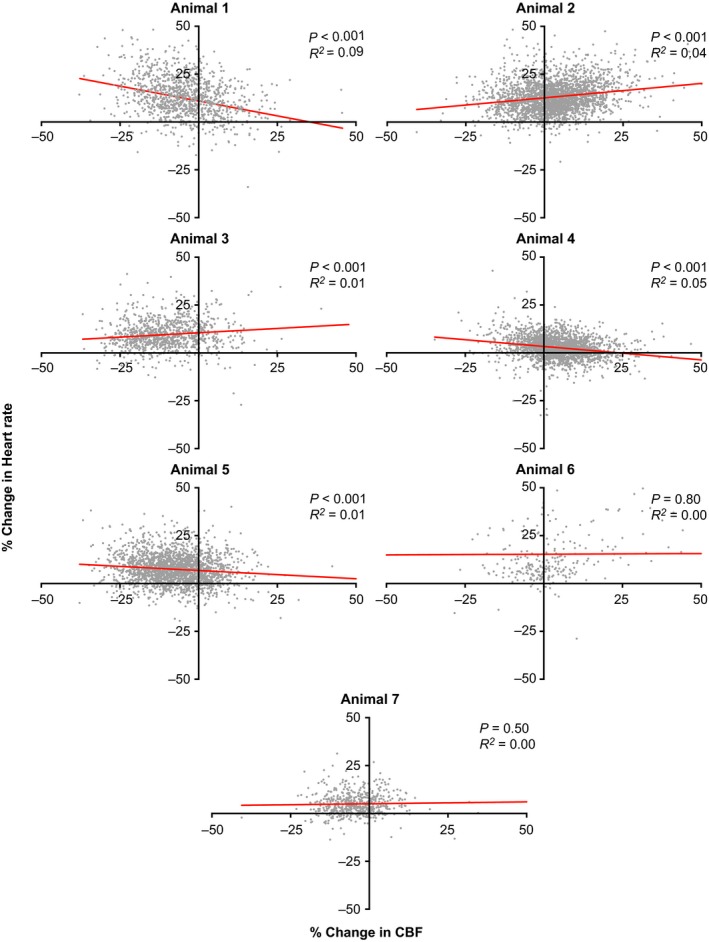
The relationship between the percent changes in heart rate and CBF (from baseline, segment 1) during segment 3. Data from each animal are shown in a separate panel; red lines show a best‐fit of the data from a linear regression analysis. The total number of data points plotted in each panel is shown in Table 1 (in the column “total number of 60 trials”). The *P*‐value indicating whether the slope was significantly different from non‐zero (*F* test) and the coefficient of variation showing the goodness of fit of the linear regression with the data are provided in each panel. These findings show that the relationships between changes in heart rate and CBF during tilts were weak, and inconsistent between animals.

**Figure 5:**
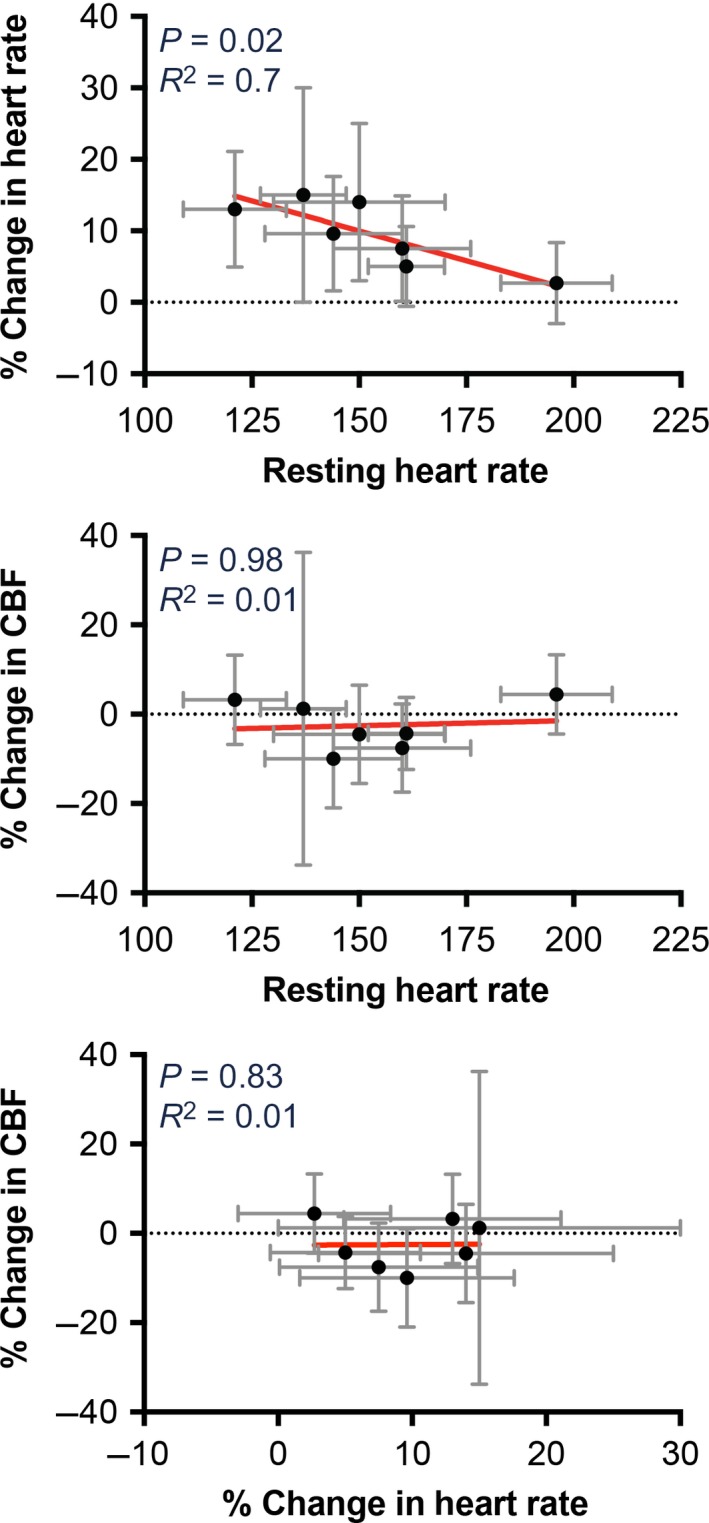
The relationship between heart rate and CBF. Each data point designates average values from an animal. Both horizontal and vertical error bars indicate one standard deviation. Red lines show a best‐fit of the data from a linear regression analysis. *P* values indicating whether the slope was significantly different from non‐zero (*F* test) as well as *R* ^2^ values showing the goodness of fit of the linear regression with the data are provided in each panel. The *top panel* compares average resting (baseline) heart rate (segment 1) with the average percent change in heart rate from baseline during recording segment 3. The middle panel compares average resting heart rate with the average percent change in CBF during recording segment 3. The bottom panel compares the average percent change in heart rate and the average percent change in CBF during segment 3. These findings show that there were no apparent relationships between resting heart rate and the change in CBF during tilts (middle panel), or the changes in heart rate and CBF during tilts (bottom panel). However, there was an inverse relationship between resting heart rate and the change in heart rate during tilts (top panel).

Figure 2 additionally illustrates that the tilt‐elicited variations in heart rate and CBF from trial‐to‐trial were very large. For instance, although on average heart rate increased during head‐up tilts, during some trials it decreased. We thus sought to determine whether alterations in heart rate and CBF during tilts were related. Figure 4 plots the relationship between the change in heart rate and CBF from baseline during segment 3 for every animal. Although the slope of the relationship was significantly non‐zero for five of seven animals, the coefficient of variation for every linear regression was less than 0.1, indicating that alterations in CBF do not explain most of the variance in heart rate. Figure 5 shows the relationships between heart rate and CBF average data of each animal. There was no apparent association between the change in CBF during segment 3 and either baseline heart rate prior to tilt (middle panel) or the change in heart rate from baseline during segment 3 (bottom panel). However, the top panel of Figure 5 indicates an association (*R* ^2 ^= 0.7) between baseline heart rate (segment 1) and the change in heart rate during tilt (segment 3), such that animals with high resting heart rates exhibited small tilt‐elicited increases in heart rates.

**Figure 6:**
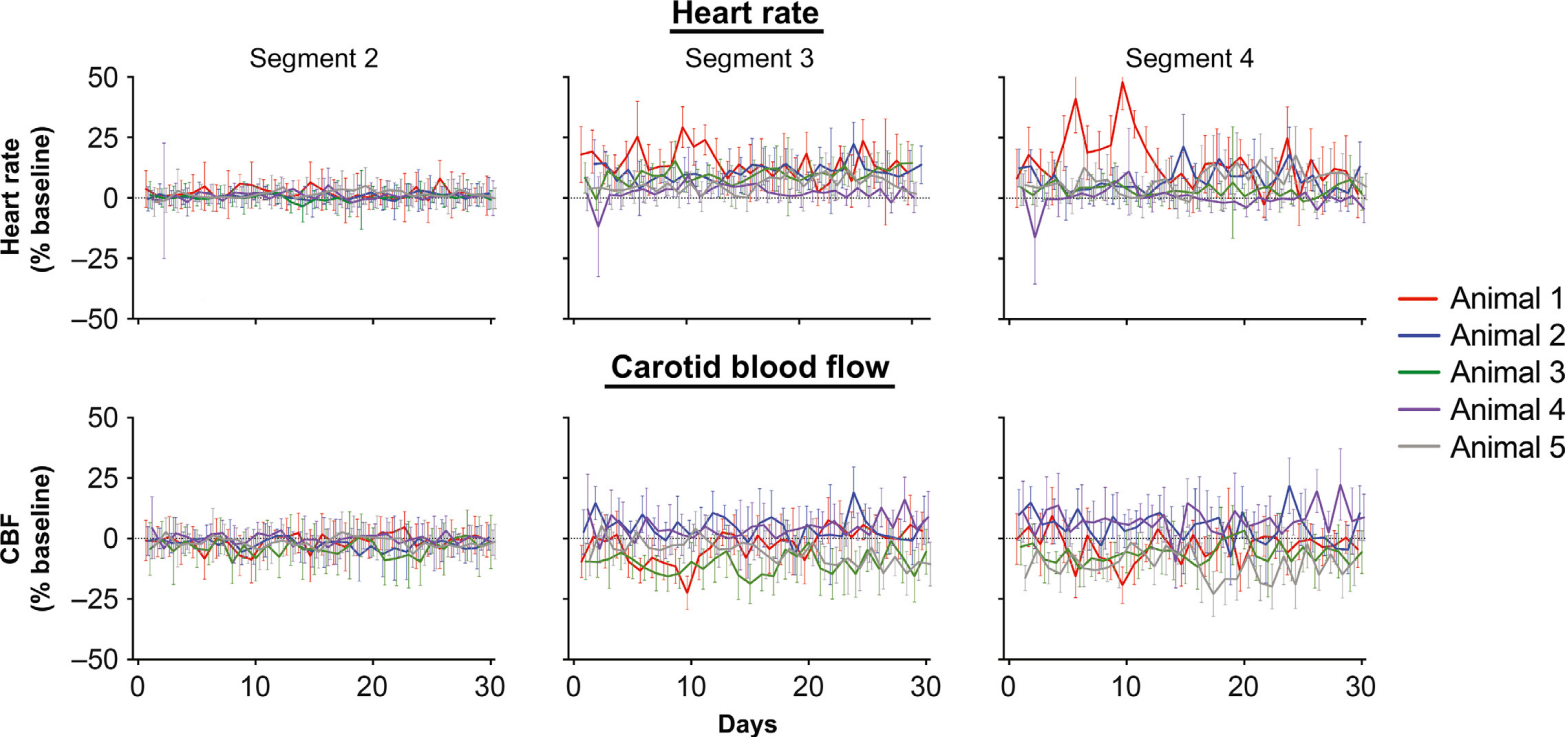
The mean daily percent change in heart rate (top panels) and CBF (bottom panels) from baseline (segment 1) in segments 2–4 during the first 30 days of testing. Data from each animal are designated by lines of different colors; data for two animals tested for less than 30 days are not shown. Error bars indicate one standard deviation. Typically, ~30 trials were conducted each day, although a few trials were eliminated from averages if animal movement or vocalization were noted, or the tilt velocity and timing did not meet standard parameters (as discussed in Methods). These findings show that there were no systematic changes in responses over time.

**Figure 7:**
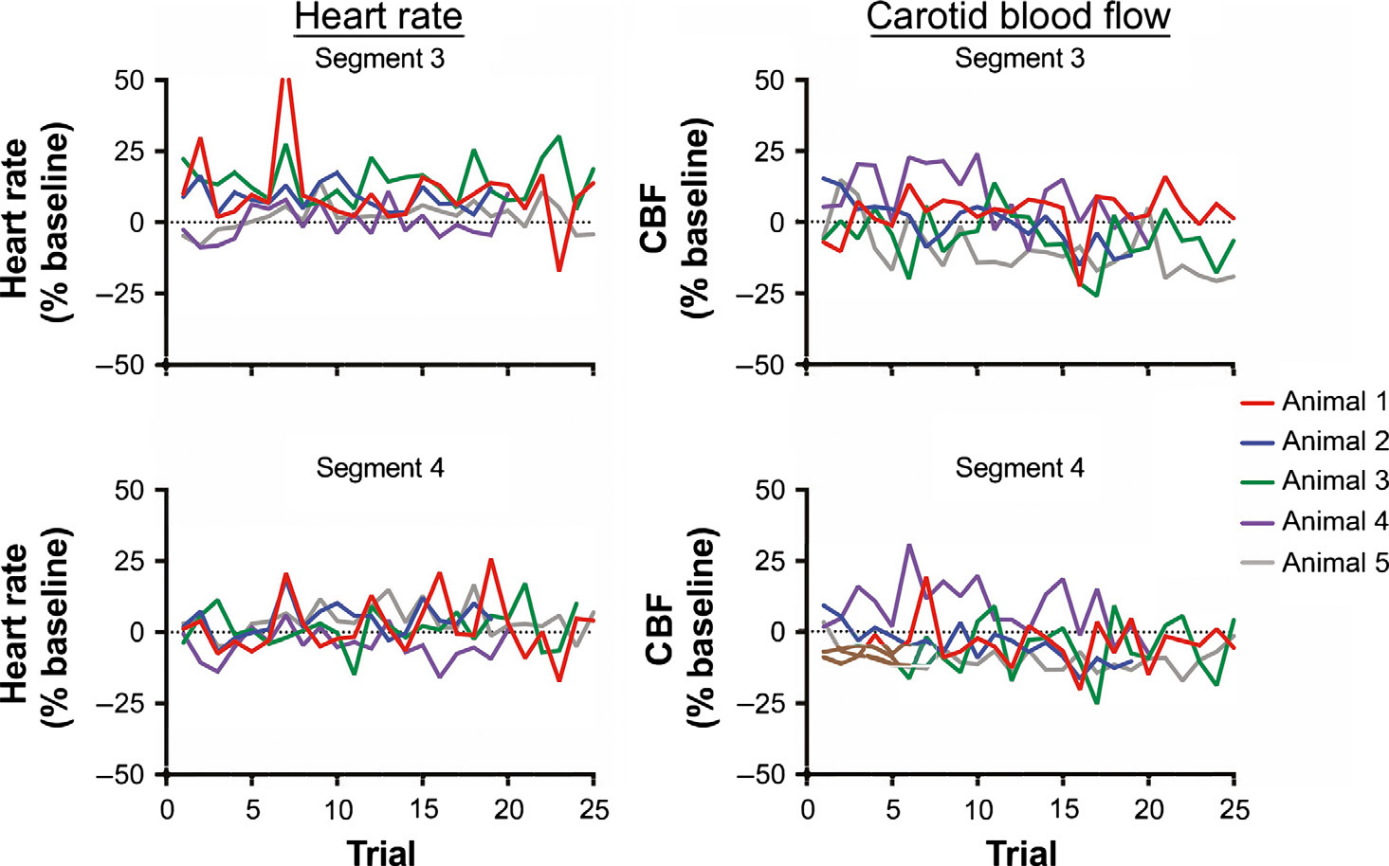
The percent changes from baseline (segment 1) in heart rate (left panels) and CBF (right panels) that occurred in each successive trial during the 30th day of testing. Changes in parameters during segment 3 are indicated in the top panels, while those in segment 4 are shown in the bottom panels. Data from the first 25 trials performed are plotted; findings in each animal are indicated by lines of different colors. These findings show that there were no systematic changes in responses over the course of a single testing session.

**Figure 8:**
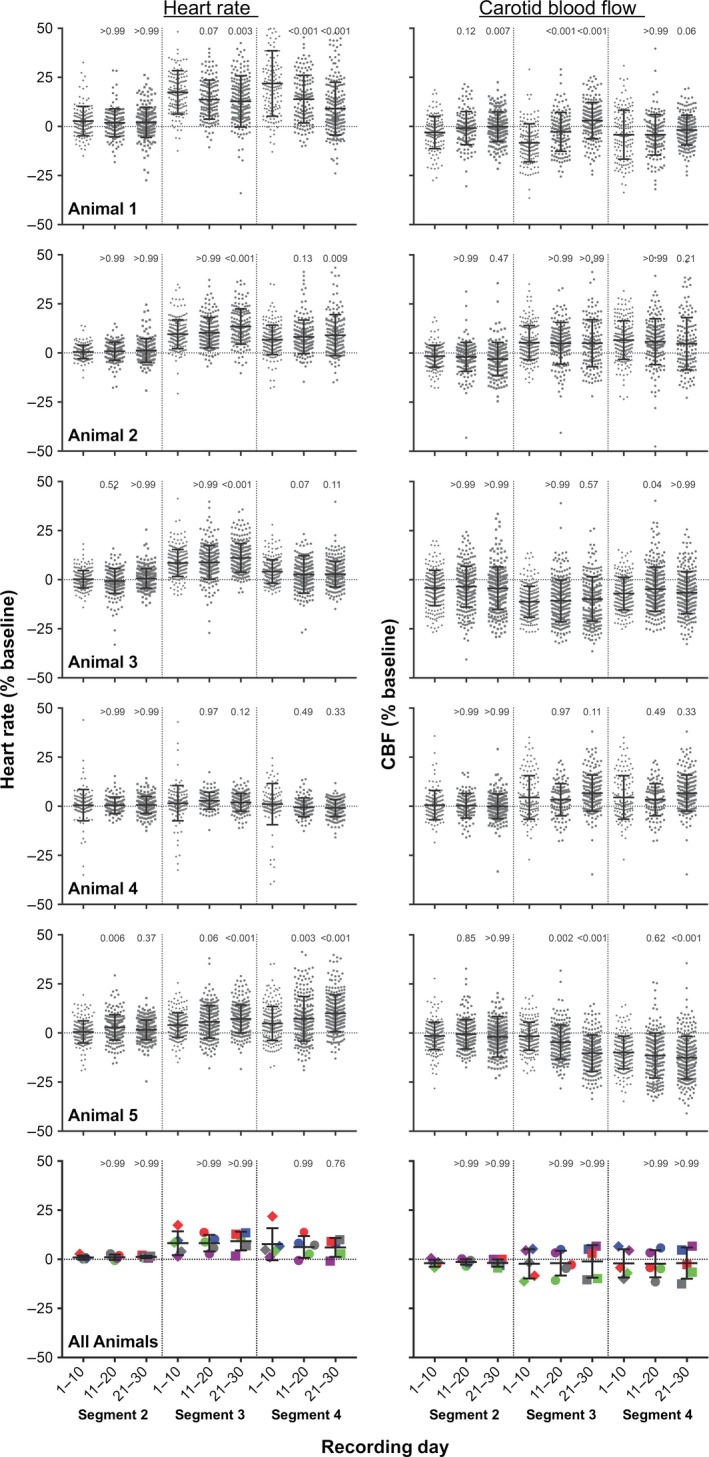
The percent change from baseline (segment 1) in heart rate (left panels) and CBF (right panels) during periods of 10 recording days (i.e., data for days 1–10, 11–20, and 21–30 were binned for analysis). Vertical dashed lines in each panel separate data for each recording segment. Numbers above each group of symbols are *P*‐values (from a two‐way ANOVA combined with Bonferroni's correction; factors were recording segment and period) designating whether the average values for days 11–20 or 21–30 were significantly different from those for days 1–10 in each recording segment. The top 5 rows illustrate data for individual trials in each animal, whereas the bottom row shows pooled data from all the animals (the average for a recording segment in an animal is designated by a single data point). In the bottom row (average results), data for each animal are designated by symbols of different colors: animal 1, red; animal 2, dark blue; animal 3, green; animal 4, purple; animal 5, gray. Error bars indicate one standard deviation. These findings complement those in Figure 6, showing that there were no systematic, statistically significant changes in responses over time.

We also considered whether tilt‐elicited alterations in heart rate and CBF changed over time, as animals became acclimated to cued 60° head‐up rotations. Figure 6 indicates the mean daily alteration in heart rate and CBF from baseline in segments 2–4 during the first 30 days of testing (data for two animals tested for less than 30 days are not shown). For segments 3 and 4, standard deviations for each day's trials were generally large, highlighting the response variability over a single 30‐min testing session. In addition, there were no apparent systematic changes in responses during the session. This response variability is further illustrated in Figure 7, which indicates the changes in heart rate and CBF that occurred in each successive trial during the 30^th^ day of testing for each animal. Figure 8 shows the average change in heart rate and CBF from baseline during periods of 10 recording days (i.e., data for days 1–10, 11–20, and 21–30 were binned for analysis). A two‐way ANOVA combined with the Bonferroni correction was used to compare the changes in heart rate and CBF for each recording segment during each subsequent 10‐day recording period (factors were recording segment and period). It is evident from Figures 6 and 8 that little change in heart rate or CBF occurred after the light cue but before the tilt (segment 2). In addition, at least for animals 2–4, the tilt‐related changes in heart rate and CBF were consistent across time in both segments 3 and 4. In animal 1, there was some evidence that over time, the change in heart rate during tilts was smaller, and there was a smaller drop in CBF. In animal 5, opposite tilt‐related changes in heart rate and CBF were noted: the increase in heart rate in both segments 3 and 4 became larger over time, while CBF declined more. When the findings from all animals were pooled (bottom panels of Fig. 8), there was no indication that the changes in heart rate or CBF during tilts differed over time.

There was also no evidence that trial‐to‐trial variability in responses decreased over time. For example, for four of the five animals, the standard deviation of heart rate changes in segment 3 was larger during days 21–30 than during days 1–10. For segment 4, the standard deviation of heart rate changes was larger in days 21–30 for three of the animals. An analysis of CBF data provided similar findings: the standard deviation of the change in CBF from baseline during both segments 3 and 4 was larger in days 21–30 than for days 1–10 in three of the five animals.

**Figure 9:**
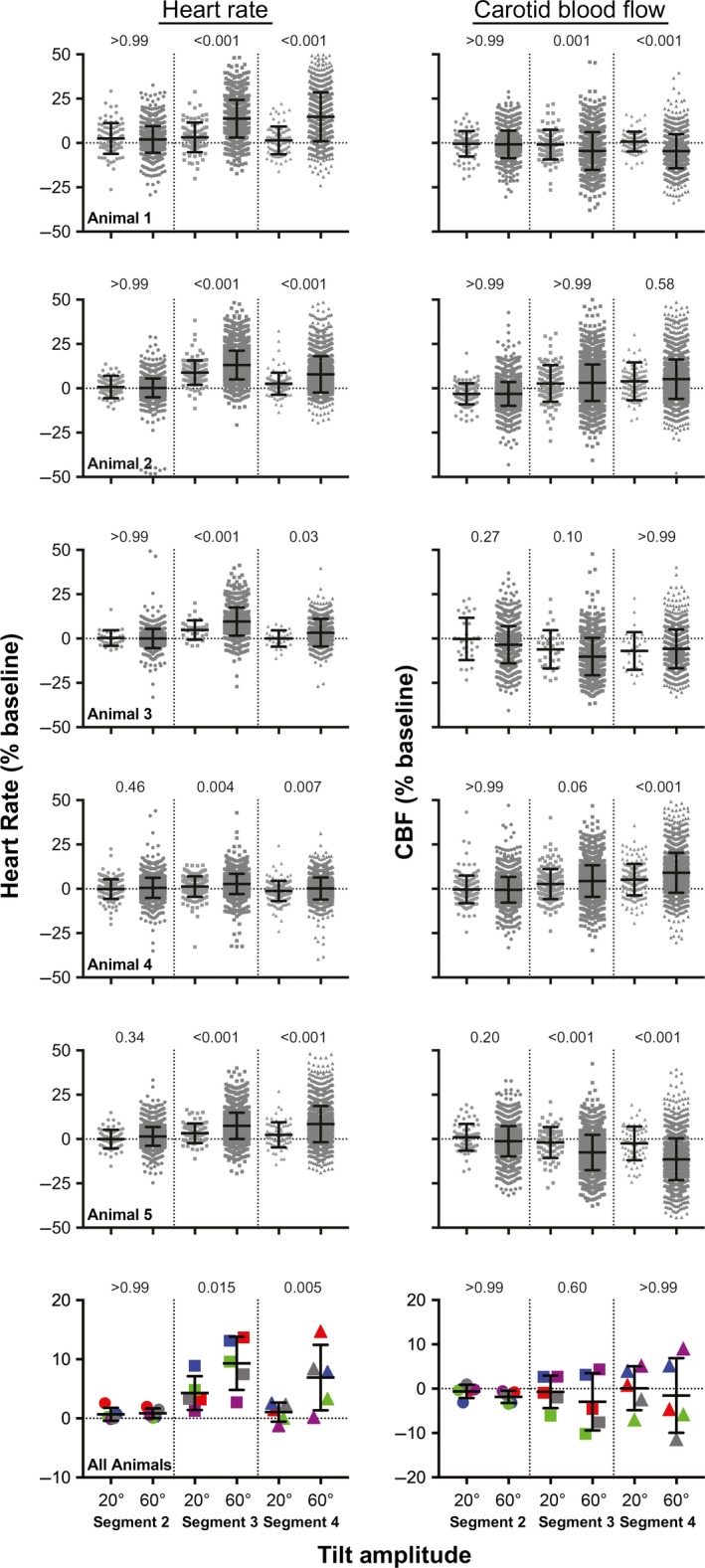
Comparison of percent changes from baseline (segment 1) in heart rate (left panels) and CBF (right panels) during 20° and 60° head‐up tilts. Vertical dashed lines in each panel separate data for each recording segment. Numbers above each group of symbols are *P*‐values (from a two‐way ANOVA combined with Bonferroni's correction; factors were recording segment and tilt amplitude) designating whether the average values for 20° and 60° tilts differed for each recording segment. The top 5 rows illustrate data for individual trials in each animal, whereas the bottom row shows pooled data from all the animals (the average for a recording segment in an animal is designated by a single data point). In the bottom row (average results), data for each animal are designated by symbols of different colors: animal 1, red; animal 2, dark blue; animal 3, green; animal 4, purple; animal 5, gray. Error bars indicate one standard deviation. The number of 20° and 60° tilts performed in each animal is provided in Table 1. These findings show that responses to 20° tilts were not exaggerated, although animals expected that 60° rotations would occur during these trials.

After delivery of cued 60° tilts for 36–64 days (see Table 1), during <10% of trials a 20° tilt was pseudorandomly substituted for the 60° rotation. The findings from this study are provided in Figure 9. A two‐way ANOVA combined with Bonferroni's correction was used to compare the changes in heart rate and CBF elicited by the two tilt amplitudes for each recording segment (factors were recording segment and tilt amplitude). Consistent with the findings discussed above, there was little change in heart rate or CBF following the light cue and prior to 20° tilts (segment 2). However, during segments 3 and 4, heart rate increased significantly more during 60° tilts than during 20° tilts in every animal. The change in CBF during 20° false‐cued tilts was in the same direction as during 60° tilt (e.g., if CBF dropped during 60° tilts, it also decreased during 20° tilts), but the magnitude of the change was either significantly smaller (animals 1, 4, and 5) or similar (animals 2 and 3) during 20° tilts. In no case was a large increase in CBF noted during 20° tilts, although animals expected that a 60° rotation would occur.

## Discussion

The primary hypothesis tested by this study was that feedforward cardiovascular responses occur prior to an anticipated passive postural change. Our experimental data do not support this hypothesis. Well‐defined changes in heart rate or CBF did not occur following a visual cue that preceded a 60° head‐up tilt, even after these stimuli were repeated over a thousand times. For most animals, although alterations in heart rate and CBF during tilts were variable from trial‐to‐trial, there were no systematic changes in the responses over time as animals became acclimated to the testing paradigm. We hypothesized that substitution of 20° for 60° tilts in a subset of trials would result in exaggerated cardiovascular responses (as animals expected 60° tilts), which were not observed. Cumulatively, these data suggest that at least in a feline model, cardiovascular adjustments to cued passive postural changes are mainly due to feedback (reflex) mechanisms, without a significant feedforward component. A caveat, however, is that posturally related hemodynamic responses are patterned, and differ in the upper and lower body (Kerman et al. 2000a,b; Wilson et al. 2006b). Thus, it is possible that the experimental paradigm used in this study elicited feedforward changes in physiological parameters that were not measured (e.g., blood flow to the hind limbs). Another caveat is that blood flow was measured from the common carotid artery, which perfuses both the brain (via the internal carotid artery) and other structures in the head (via the external carotid artery). Although most of the common carotid blood flow is directed to the brain (Sato et al. 2012), it is possible that anticipation of movement generates distinct changes in external and internal carotid blood flow that confounded the interpretation of the findings from this study. Such disparities in external and internal carotid blood flow have been documented during hypotension and exercise (Sato et al. 2011; Ogoh et al. 2014).

It is well‐established that feedforward cardiovascular responses occur at the onset of exercise (Waldrop et al. 1996; Fadel and Raven 2012; Fisher et al. 2015). The movement paradigm in this study differed in two major ways from that during exercise: (1) the change in body position was passive, and did not involve muscle contraction and (2) the change in body position did not require the extensive and prolonged cardiovascular responses that occur during exercise. Gandevia's findings that imagined exercise in paralyzed human subjects produce large changes in heart rate and blood pressure indicate that muscle contraction is not needed to evoke feedforward cardiovascular responses related to movement (Gandevia et al. 1993). However, in that study movement was contemplated, while in the present experiments animals presumably did not attempt to move. Thus, the planning of active movement may be key to eliciting feedforward cardiovascular responses, which are absent when movements are imposed. Although this notion is yet to be tested experimentally, it may have practical implications. For example, it is possible that orthostatic hypotension during head‐up tilting of bedridden patients could be avoided if the patients imagine standing during the passive movement.

Another possibility yet to be examined is that feedforward cardiovascular responses to movement only occur if the intended movement requires large changes in cardiac output. Perhaps the modest increases in heart rate and total peripheral resistance that accompany head‐up tilts (Blomqvist and Stone 1983; Wilson et al. 2006b; Yates et al. 2014; Ricci et al. 2015) do not reach the threshold for necessitating feedforward cardiovascular responses. Conditioned cardiovascular responses are strongest when they are associated with large unconditioned stimuli (Cohen and Randall 1984; McEchron et al. 1992; Maren 2001), such that feedforward cardiovascular responses during movement might only occur when the movement requires large adjustments in sympathetic nervous system activity. As such, movement‐related feedforward cardiovascular responses might be unique to exercise, or situations where the unconditioned stimulus is more salient than employed in this study (e.g., a stimulus that produces fear) (Maren 2001).

Although previous studies characterized changes in CBF during head‐up tilts (Wilson et al. 2006a), this study provided the most comprehensive longitudinal analysis that we are aware of about the effects of postural changes on carotid blood flow. Notably, the intrasubject alterations in heart rate and CBF during tilts were highly variable, including during a single testing session, and in most animals this variability did not decline over time. They did not apparently adapt to the stimuli by adjusting autonomic or motor responses (e.g., tensing body muscles during rotations). Intersubject variability in cardiovascular responses to passive head‐up tilts has been reported in human subjects, although repetition of testing in these studies was not nearly as extensive as in the present one (Vaz et al. 1991; Petersen et al. 2000); for review see (Blomqvist and Stone 1983). Thus, the present study contributes to the literature by highlighting the considerable intrasubject variability in orthostatic responses.

As discussed above, feedback mechanisms appear to be the major contributor to regulating cardiovascular responses to passive head‐up tilts. The data from this study support the notion that these feedback mechanisms are multifaceted. Changes in heart rate and CBF during 60° tilts were not correlated, such that CBF could drop during large increases or decreases in heart rate, and vice‐versa. Thus, alterations in sympathetic nervous system activity were not the apparent primary determinants of response variability. Responses over a single recording session were inconsistent, indicating that factors that change slowly (such as hydration) were not the principal contributor to intrasubject variability in heart rate and CBF responses to tilts. Although animals were observed to be awake during all trials, fluctuations in their vigilance could have contributed to response variability. Both vestibular and visual sensory cues are utilized to achieve blood pressure stability during passive head‐up tilts (Jian et al. 1999), and the processing of these inputs is altered by vigilance, as evidenced by degraded postural control in subjects that are distracted (Mahboobin et al. 2007). Further studies of the etiology of intrasubject variability in orthostatic cardiovascular responses is warranted, as they may provide insights into methods to decrease posturally related fluctuations in head blood flow in patients who are susceptible to orthostatic hypotension. Other potential contributors to variations in head blood flow could have been diversity in tilt‐elicited respiratory changes (Arshian et al. 2017) or tensing of muscles that affected venous return to the heart and thus cardiac output.

In summary, several lines of evidence from this study showed that use of a light cue to signal a 60° head‐up tilt did not elicit feedforward cardiovascular responses that stabilize carotid blood flow. Instead, feedback mechanisms appear to play the primary role in eliciting cardiovascular responses to passive (imposed) changes in posture. However, there was considerable intrasubject and intersubject variability in CBF and heart rate during head‐up tilts, indicating inconsistencies in the execution of these feedforward mechanisms. Further work is needed to ascertain the etiology of this response variability, including variations in responses that occur over a single testing session.

## Conflict of Interest

None declared.
